# Correlation of the differential expressions of RANK, RANKL, and OPG with obesity in the elderly population in Xinjiang

**DOI:** 10.1515/med-2025-1196

**Published:** 2025-08-07

**Authors:** Jinling Liu, Wenwen Xiao, Buluhan Halan, Aishanjiang Wumaer, Zhuoya Maimaitiwusiman, Saiyare Xuekelati, Tajiguli Musha, Xue Bai, Hongmei Wang

**Affiliations:** The Second Ward of the Health Center for Cadre of People’s Hospital of Xinjiang Uygur Autonomous Region, Urumqi, 832000, China

**Keywords:** elderly population, obesity, RANK, RANKL, OPG

## Abstract

**Objective:**

Obesity has been recognized as a global epidemic and public health crisis. The prevalence of obesity has tripled since 1975, especially in the elderly population. Accumulating preclinical and clinical studies have confirmed that chronic low-grade inflammation of adipose tissues is mechanistically related to metabolic diseases and tissue complications in overweight and obesity patients, as a result of the intricate interplay of various pro-inflammatory and anti-inflammatory signaling cascades. The present study aims to explore the differential expressions of receptor activator of nuclear factor-kappa B (RANK), RANK ligand (RANKL), and osteoprotegerin (OPG) in leukocytes of elderly obesity patients in Xinjiang, thereby providing new physiological, cellular, and molecular targets for the treatment of obesity.

**Methods:**

A total of 40 obesity patients and 40 non-obesity patients were selected. The protein and gene expression levels of RANK, RANKL, and OPG in peripheral blood were detected by western blotting and real-time quantitative PCR.

**Results:**

There was no significant difference in the expression levels of RANK protein, OPG protein, and OPG gene between the obesity group and non-obesity group (*P* > 0.05). The relative expression levels of the RANK gene and RANKL protein and gene were significantly different between the obesity group and non-obesity group (*P* < 0.05). The differential expressions of the RANK gene, RANKL protein and gene, and the OPG protein in leukocytes had a significant correlation with obesity in the elderly population (*P* < 0.05).

**Conclusion:**

The expressions of the RANKL protein and gene and RANK gene in leukocytes of elderly obesity patients in Xinjiang were higher than those of non-obesity patients. In the elderly obesity patients in Xinjiang, the abdominal circumference was correlated with the expressions of the RANK gene, RANKL protein and gene, and OPG protein in leukocytes.

## 1 Introduction

Obesity is defined as a chronic metabolic disease caused by excessive fat accumulation and also includes genetic and environmental factors [1]. Obesity can be asymptomatic or accompanied by a variety of comorbidities, such as cancer, coronary artery disease, diabetes, hypertension, gout, obstructive sleep apnea, and osteoarthritis [2,3,4]. It is estimated that nearly 4 million people die of weight-related comorbidities every year. Complications caused by obesity are closely related to the risk of death and have become the primary cause of preventable diseases and disabilities [5]. According to the survey, 2030–2050 will be the most serious period of population aging in China. The global obesity epidemic continues to spread relentlessly. In China, the elderly over 60 years old are particularly prominent, which leads to an increase in the risk of serious diseases and death related to obesity and its comorbidities. It is easy to interact with other risk factors, further endangering the health of the elderly. Therefore, it is imperative to formulate and implement effective obesity prevention and control strategies [6].

Obesity is the result of the complex interaction of genetic factors, environmental factors, and polygenic factors. At present, research on the pathogenesis of obesity is still in the exploratory stage, and the exact cause of obesity has not been fully clarified. New evidence shows that the nuclear factor receptor activator-κB ligand (RANKL)-RANK-OPG system not only participates in the regulation of bone metabolism but also plays a vital role in the regulation of chronic metabolic diseases, especially sugar and lipid metabolism [7,8]. The variation and differential expression of these genes are partially the reasons for obesity [7,8,9,10,11,12,13,14,15]. Our previous research found that the decrease in the methylation level in the RANK promoter region is related to obesity in the elderly population in Xinjiang [16]. Other scholars have found that there is an obvious correlation between the reduction of the methylation level and obesity in the population, and pointed out that the effect is very significant, especially in the elderly over 65 years old. However, there is no report on the correlation between the gene and protein expression levels of the Rank–RANKL–OPG system and obesity. Relevant scholars have pointed out that RANKL binds to its receptor level and activates the NF-κB pathway, thus triggering the expression of pro-inflammatory cytokines, while obesity also activates the JNK and NF-κB signaling pathways. NF-κB activation induces the activation of the inflammatory signaling pathway, which leads to insulin resistance and pancreatic β-cell dysfunction, and also activates T cells, endothelial cells, and adipocytes. By comparing the gene and protein expression levels of RANK, RANKL, and OPG in elderly obese patients in Xinjiang, this study discusses the correlation between the RANK–RANKL–OPG system and elderly obesity, and provides a basis for further study on the pathogenesis of obesity.

## 2 Research subjects and methods

### 2.1 Research subjects

The cohort established in this study was based on the elderly general population in Xinjiang collected in the previous epidemiological survey. According to the Guidelines for Prevention and Control of Overweight and Obesity in Chinese Adults, a male waist circumference of ≥85 cm and a female waist circumference of ≥80 cm were considered obese. A total of 80 older patients who were hospitalized in the Second Department of Cadre Health Care of People’s Hospital of Xinjiang Uygur Autonomous Region from March 2020 to April 2020 were recruited. Inclusion criteria are as follows: (1) age ≥ 60 years, (2) local registered residents, and (3) informed consent. Exclusion criteria are as follows: (1) cognitive impairment, (2) language communication barrier, (3) patients with mental illness, (4) type I diabetes, (5) chronic kidney disease, (6) chronic liver disease or alcoholism, (7) chronic lung disease, (8) taking hormone drugs, (9) hyperthyroidism and hypothyroidism, (10) major organ failure, (11) a recent history of surgery, (12) a recent history of infection, and (13) consumptive diseases such as malignant tumor and pulmonary tuberculosis.

The general information of all subjects was collected, including the age, gender, educational degree, resting blood pressure, smoking and drinking history, history of diabetes, and history of hypertension. Then, 5 mL of whole blood was extracted from the subjects under fasting conditions, and the serum and blood-formed elements were separated by on-site centrifugation. The blood lipids were detected within 1 month after blood collection, including total cholesterol (TC), triglyceride (TG), high-density lipoprotein-cholesterol (HDL-C), low-density lipoprotein-cholesterol (LDL-C), creatinine, glutamic pyruvic transaminase, glutamic oxaloacetic transaminase, blood glucose, and other biochemical indicators. The blood-formed element was stored at −80°C for extracting DNA. The biochemical tests were completed by specially assigned personnel in the Department of Clinical Laboratory of the People’s Hospital of Xinjiang Uygur Autonomous Region (grade III class A hospital).

The calculation of each index was as follows: (1) hypertension: systolic pressure ≥ 140 mmHg (1 mmHg = 0.133 kPa) and/or diastolic pressure ≥ 90 mmHg, or those who had a history of hypertension and took antihypertensive drugs in the past 2 weeks [17]. (2) Diabetes: fasting blood glucose (FBG) ≥ 7.0 mmol/L and/or with a history of diabetes [18]. (3) Dyslipidemia: TC ≥ 6.22 mmol/L or TG ≥ 2.26 mmol/L or LDL-C ≥ 4.14 mmol/L or HDL-C < 1.04 mmol/L [19]. (4) Abdominal circumference: the subject was in a standing position, with shoulders naturally relaxed and arms sagging. A tape measure was used to encircle the navel of the subject, and the tape measure should be parallel to the ground when recording the abdominal circumference. Men with an abdominal circumference of ≥85 cm and women with an abdominal circumference of ≥80 cm were considered obese.

Among the selected subjects, there were 40 subjects in the obesity group (case group) and 40 subjects in the non-obesity group. All subjects had signed informed consent. This study was reviewed and approved by the Medical Ethics Committee of the People’s Hospital of Xinjiang Uygur Autonomous Region.

### 2.2 Research materials

Five × All-In-One RT MasterMix (with AccuRT Genomic DNA Removal Kit) (abm, G492), EvaGreen Express 2 × qPCR MasterMix-Low Rox (abm, MasterMix-LR), bicinchoninic acid (BCA) kit (TransGen Biotech, DQ111-01), RANK antibody (Abcam, ab182158), RANKL antibody (Bioss, bs-0747R), OPG antibody (Bioss, bs-0431R), LC3-B antibody (Bioss, BS-4843R), and β-actin (Sino Biological, 100166-MM10) were used in the experiments.

A PCR instrument (Bio-Rad, MyCycler Thermal Cycler, ABI QuantStudio™ 6), quantitative fluorescence PCR (ABI, Flex Real-Time PCR System), high-speed refrigerated centrifuge (Thermo Fisher Scientific, Heraeus Multifuge X1R), gel imaging system (Shanghai Tanon Co., Ltd, 2500), electrophoretic transfer (Bio-Rad, USA, Mini-PROTEAN Tetra System), chemiluminescence imaging system (Shanghai CLiNX Scientific Instruments Co., Ltd, Chemiscope 3000), and microplate reader (Bio-Rad, xMarkTM) were used.

### 2.3 Research methods

#### 2.3.1 Treatment of blood samples

Blood samples were collected from the subjects using ethylenediaminetetraacetic acid (EDTA) tubes (5 mL × 2 tubes per subject) and centrifuged at 2,500 rpm/min (centrifugation radius of 116 mm) for 10 min to obtain the upper plasma and sealed it for standby. The remaining lower blood sample was added with three times the volume of red blood cell lysate, mixed twice in a vortex shaker, and placed on ice for 15 min. The sample was subjected to centrifugation at 450 × *g* and 4°C for 10 min to precipitate leukocytes, and then, the supernatant was carefully removed. Next, the leukocyte precipitation was supplemented with red blood cell lysate, twice the volume of the original liquid, followed by a gentle vortex to fully resuspend the leukocytes. Thereafter, the leukocytes were centrifuged at 450 × *g* and 4°C for 10 min, and the supernatant was removed carefully and thoroughly. Finally, peripheral blood leukocytes were obtained.

#### 2.3.2 Extraction of protein and RNA

Leukocytes (1 × 10^6^) were collected, rinsed with phosphate-buffered saline (PBS), and centrifuged to remove the supernatant. To the cell precipitate, 100 μL radioimmunoprecipitation assay (RIPA) buffer containing protease inhibitor and phosphatase inhibitor was added, homogenized fully on ice, subjected to an ice bath for 60 min, and centrifuged at 12,000 rpm and 4°C for 15 min to obtain the supernatant. The protein concentration was measured using the BCA method. Subsequently, the protein was denatured with 5× protein loading buffer and then stored for standby after cooling.

Peripheral blood leukocytes were lysed with the TRIzol reagent, mixed in a 1.5 mL centrifuge tube, and placed at room temperature for 15 min. Then, the cells were mixed with 200 μL of chloroform, placed at room temperature for 5 min, and centrifuged at 12,000 rpm and 4°C for 15 min. The supernatant was moved into another centrifuge tube, an equal volume of isopropanol was added, mixed well, and placed at −20°C for 60 min. After centrifugation at 12,000 rpm for 15 min at 4°C, the supernatant was carefully removed. Next, 75% ethanol was added to make the precipitate float and washed off. After centrifugation at 12,000 rpm for 15 min at 4°C to remove the supernatant, 75% ethanol was added again for washing, followed by empty tube centrifugation and discarding the liquid. The precipitate was dissolved using RNase-free water. The ultramicrospectrophotometer (Nano-100, ALLSHENG, Hangzhou, China) was used for the detection. The final concentration of RNA in each sample was ensured to be 100–500 ng/μL, and the A260/A280 ratio was 1.8–2.1. RNA integrity was detected using agarose gel electrophoresis, followed by RNA reverse transcription for subsequent experiments.

#### 2.3.3 Western blotting

The loaded protein sample (20 μg) was separated by 10% sodium dodecyl sulfate-polyacrylamide gel electrophoresis (SDS-PAGE) and then transferred onto polyvinylidene fluoride membranes (0.45 μm). The membranes were blocked with 5% skim milk at room temperature for 1 h, rinsed with Tris-buffered saline-tween (TBST) 3 times (5 min/time), and incubated with the primary antibodies at 4°C overnight: RANK (1:1,200), RANKL (1:1,600), OPG (1:600), and β-actin (1:1,000). After washing three times with TBST (10 min/time), the membranes were incubated with the secondary antibodies goat anti-mouse IgG H&L (HRP) (1:15,000) and goat anti-rabbit IgG H&L (HRP) (1:5,000) at room temperature for 1 h and rinsed with TBST three times (10 min/time). The chromogenic solutions A and B were mixed and added to the membranes (2 mL), and the ChemiScope mini chemiluminometer was used for detection and photographing. The gray value of protein bands was calculated using Image J software.

#### 2.3.4 cDNA synthesis and quantitative real-time polymerase chain reaction (qPCR)

The reverse transcription system is shown in Table 1. Reverse transcription was performed using the PCR instrument (Bio-Rad, MyCycler Thermal Cycler, ABI QuantStudio™ 6), and the procedures were set as follows: reaction at 25°C for 10 min, reaction at 42°C for 15 min, and reaction at 85°C for 5 min. The cDNA obtained by reverse transcription was stored at −80°C, and the cDNA samples for subsequent fluorescence quantitative detection of mRNA were diluted with RNase-free water at a ratio of 1:1 for the reaction. The primer information is shown in Table 2: β-actin, internal reference, 10 μL Evagreen 2 × qPCR Master Mix (abm, MasterMix-LR); 0.6 μL, forward primer; 0.6 μL, reverse primer; 2 μL, cDNA template; and 6.8 μL, ddH_2_O. The procedures of the fluorescence quantitative PCR instrument were set as follows: 95°C for 10 min, 40 cycles of 95°C for 15 s, 60°C for 60 s, and 60°C for 60 s. The relative expression of the gene was calculated using the 2^−ΔΔ*C*t^ method.

**Table 1:** Synthesis and configuration system of mRNA first-strand cDNA

Component	Volume
RNA template	Up to 1 μg
AccuRT reaction mix (4×)	2 μL
Nuclease-free H_2_O	Up to a total volume of 8 μL
Incubate at 42°C for 2 min or at room temperature for 5 min, then add the following to the tube:	
AccuRT Reaction Stopper (5X)	2 μL
5X All-In-One RT MasterMix	4 μL
Nuclease-free H_2_O	6 μL
Total reaction volume	20 μL

**Table 2:** Primers for fluorescence quantitative mRNA detection

Name of the primer	Sequence (5′ to 3′)	Size of the primer
RANK-F	TTGCAGCTCAACAAGGACAC	104 bp
RANK-R	AAGGTACAGTTGGTCCAGGG	
RANKL-F	CAGAGAAAGCGATGGTGGATG	105 bp
RANKL-R	ATGGGATGTCGGTGGCATTA	
OPG-F	AAGGAGCTGCAGTACGTCAA	139 bp
OPG-R	CAGCTTGCACCACTCCAAAT	

#### 2.3.5 Statistical methods

All data were subjected to statistical analysis by SPSS 19.0 software. The measurement data were in normal distribution and expressed by *x* ± *s*. The difference between the two groups was compared by using a *t*-test. The correlation of the expressions of the RANK, RANKL, OPG protein, and gene with obesity was analyzed by the Pearson correlation coefficient. A value of *P* < 0.05 was indicative of statistical significance.


**Ethics approval:** This study was reviewed and approved by the Medical Ethics Committee of the People’s Hospital of Xinjiang Uygur Autonomous Region.
**Informed consent:** For all studies involving human participants, informed written consent to take part in the research has been obtained prior to the commencement of the study.

## 3 Results

### 3.1 Comparison of demographic and clinical characteristics

This section includes the basic indexes and clinical characteristics of obese and non-obese groups, and analyzes the correlation between patients’ age, gender, diastolic blood pressure at admission, education level, hypertension, diabetes, coronary heart disease, TG, TC, LDL-C, HDL-C, urea nitrogen, and so on.

The analysis of Table 3 shows that there are significant differences in age, sex, and diastolic blood pressure at admission between the obese group and non-obese group, which is statistically significant. It shows that obesity and non-obesity are influenced by age, sex, and diastolic blood pressure, and many factors are closely related. The analysis found that no matter the education level of patients and the medical history of hypertension, diabetes, coronary heart disease, and other differences, there will be no significant comparative differences, no statistical significance. The same is true for TG, TC, LDL-C, HDL-C, and urea nitrogen.

**Table 3:** Analysis of demographic characteristics and clinical data of selected subjects

	Obesity group (*n* = 40)	Non-obesity group (*n* = 40)	*t*/*Z*/*χ*²	*P*
Age	72.15 (7.18)	76.97 (10.29)	2.432	0.017
**Gender (%)**			6.545	0.02
Male	20 (50.0)	31 (77.5)		
Female	20 (50.0)	9 (22.5)		
**Nationality (%)**			2.615	0.27
Han Nationality	23 (57.5)	23 (57.5)		
Uygur Nationality	11 (27.5)	15 (37.5)		
Other	6 (15.0)	2 (5.0)		
**Education degree (%)**			3.252	0.197
Primary school degree	5 (12.5)	11 (27.5)		
Junior high school degree	4 (10.0)	5 (12.5)		
Senior high school degree or above	31 (77.5)	24 (60.0)		
Smoking (%)	12 (30.0)	10 (25.0)		0.802
Drinking (%)	9 (22.5)	8 (20.0)		1
Type 2 diabetes (%)	7 (17.5)	12 (30.0)		0.293
Hypertension (%)	24 (60.0)	32 (80.0)		0.088
Coronary heart disease (%)	18 (45.0)	17 (42.5)		1
Systolic pressure at admission	138.20 (20.43)	137.15 (19.11)	−0.237	0.813
Diastolic pressure at admission	78.17 (12.64)	72.20 (13.03)	−2.082	0.041
Glutamic pyruvic transaminase	21.82 (13.26)	20.90 (19.97)	−0.244	0.808
Glutamic oxaloacetic transaminase	20.96 (6.77)	21.67 (17.23)	0.242	0.809
Urea nitrogen	4.96 (1.32)	5.70 (2.12)	1.869	0.065
Creatinine	64.90 (13.14)	76.12 (40.55)	1.665	0.1
TG	1.36 (0.52)	1.38 (0.68)	0.177	0.86
TC	4.46 (1.14)	4.39 (1.27)	−0.263	0.793
High-density lipoprotein	1.16 (0.25)	1.24 (0.69)	0.740	0.461
Low-density lipoprotein	2.03 (0.82)	2.06 (0.89)	0.124	0.902

### 3.2 Comparison of RANKL protein and RNA expression levels

**Figure 1:**
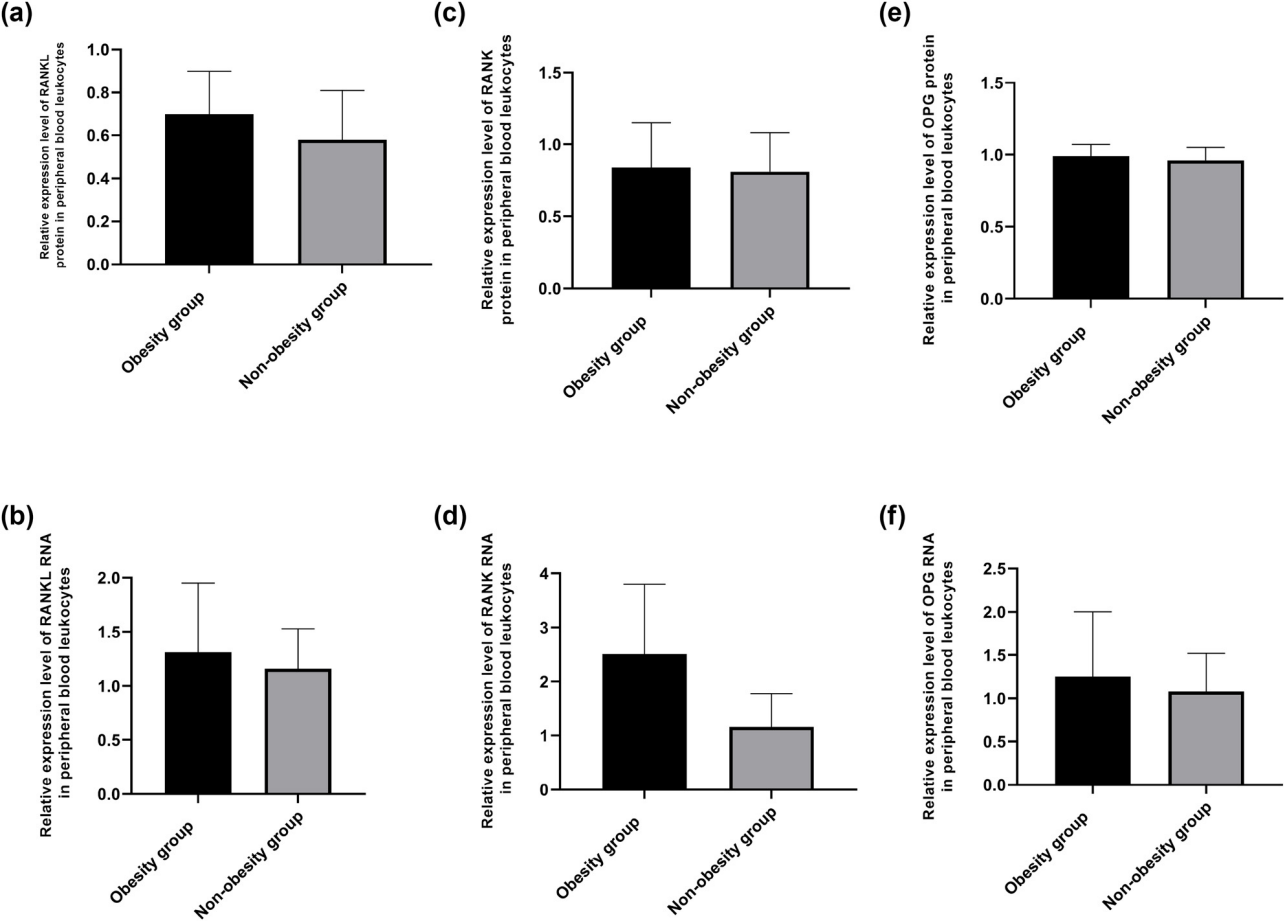
Comparison of protein levels of RANKL, RANK, and OPG in the peripheral blood between the two groups ((a) statistical analysis of RANKL protein expression level; (b) statistical analysis of RANK protein expression level; (c) statistical analysis of OPG protein expression level). Comparison of gene levels of RANKL, RANK, and OPG in peripheral blood between the two groups ((d) statistical analysis of the RANKL gene expression level; (e) statistical analysis of the RANK gene expression level; (f) statistical analysis of the OPG gene expression level).

**Figure 2:**
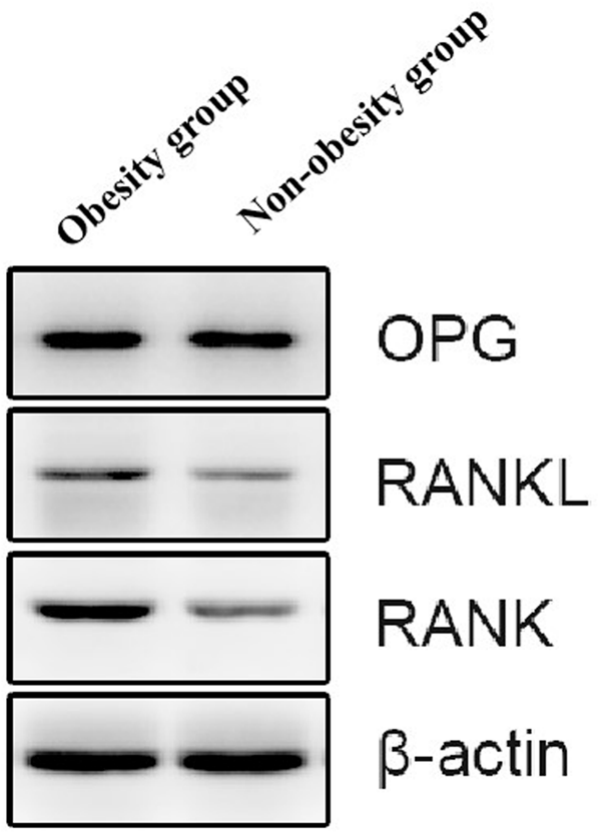
Expression levels of the RANK, RANKL, and OPG proteins in the two groups.

The relative expression of RANKL protein was significantly different between the obesity group [(0.70 ± 0.20)] and the non-obesity group [(0.58 ± 0.23)] (*T* = −2.657, *P* = 0.021), and the expression of RANKL mRNA was also significantly different between the obesity group [(1.31 ± 0.64)] and the non-obesity group [(1.06 ± 0.37)] (*T* = −2.144, *P* = 0.035), as shown in Table 4 and Figure 1. Expression levels of RANK, RANKL, and OPG proteins in two groups are shown in Figure 2.

**Table 4:** Difference in the relative expressions of RANKL, RANK, OPG proteins and mRNAs between older obese patients and controls

	Obesity group	Non-obesity group	*T*	*P*
RANKL protein	0.70 (0.20)	0.58 (0.23)	−2.657	0.021
RANKL mRNA	1.31 (0.64)	1.06 (0.37)	−2.144	0.035
RANK protein	0.84 (0.31)	0.81 (0.27)	−0.144	0.701
RANK mRNA	2.51 (1.29)	1.16 (0.62)	−5.969	<0.001
OPG protein	0.99 (0.08)	0.96 (0.09)	1.154	0.117
OPG mRNA	1.25 (0.75)	1.08 (0.44)	−1.174	0.244

### 3.3 Comparison of the RANK protein and gene expression levels

There was no significant difference in the relative expression of RANK protein between the obesity group [(0.99 ± 0.08)] and the non-obesity group [(0.81 ± 0.27)] (*T* = 0.144, *P* = 0.701), while the expression of RANK gene was significantly different between the obesity group [(2.51 ± 1.29)] and the non-obesity group [(1.16 ± 0.62)] (*T* = −5.969, *P* < 0.001), as shown in Table 4 and Figure 1.

### 3.4 Comparison of OPG protein and gene expression levels

There was no significant difference in the relative expression of OPG protein between the obesity group [(0.98 ± 0.08)] and the non-obesity group [(0.97 ± 0.09)] (*T* = 1.1541, *P* = 0.117). The expression of OPG mRNA was significantly different between the obesity group [(1.25 ± 0.75)] and the non-obesity group [(1.08 ± 0.44)] (*T* = −1.174, *P* = 0.244), as shown in Table 4 and Figure 1.

### 3.5 Correlation analysis between the differential expressions of the RANK, RANKL, OPG proteins, and gene and abdominal circumference

The expressions of the RANK mRNA, RANKL protein, RANKL mRNA, and OPG protein in leukocytes of elderly obese patients were positively correlated with abdominal circumference, and the correlation coefficient *r* was 0.378, 0.043, 0.262, and 0.363, respectively, with statistical significance (all *P* < 0.05, Table 5).

**Table 5:** Correlation between the abdominal circumference and relative expressions of the RANKL, RANK, OPG proteins, and mRNAs in older obesity patients

	Abdominal circumference	RANK protein	RANKL protein	OPG protein	RANK mRNA	RANKL mRNA	OPG mRNA
RANK protein	0.292	1.000	0.233	0.211	−0.046	−0.206	−0.066
RANKL protein	0.000	0.038	1.000	0.407	0.116	−0.093	−0.042
OPG protein	0.001	0.061	0.000	1.000	0.023	0.298	−0.103
RANK mRNA	0.001	0.684	0.306	0.838	1.000	0.177	0.099
RANKL mRNA	0.019	0.067	0.414	0.007	0.116	1.000	0.371
OPG mRNA	0.443	0.558	0.711	0.362	0.383	0.001	1.000
Abdominal circumference	1.000	−0.119	0.443	0.363	0.378	0.262	0.087

Note: Numbers in black are the Pearson correlation coefficients, and numbers in red are the *P* values of the correlation coefficients.

## 4 Discussion

The results of this study showed that the expressions of the RANKL protein, RANKL mRNA, and RANK gene in the peripheral blood of older obese patients in Xinjiang were higher than those of non-obese patients. In the older obese patients in Xinjiang, the abdominal circumference was correlated with the expression of the RANK protein, RANK gene, RANKL protein, and OPG protein. However, the interaction and mechanism between obesity and the RANK/RANKL/OPG system still need further exploration.

Nowadays, obesity has become the most concerning public health problem in the world [20,21,22]. Chronic low-grade inflammation of the whole body is an important inducement of obesity, lipid metabolism, diabetes, arteriosclerosis, and insulin resistance [23]. In addition, some researchers pointed out that adipose tissue can secrete many hormones, cytokines, and chemokines with various biological functions besides energy storage [24], thus participating in the regulation of adipose tissue and the immune system [25]. Obesity is not only the excessive accumulation of adipose tissue, but also a chronic low-grade inflammatory state induced by various inflammatory factors [25]. In the vicious circle of obesity, the continuous expansion of white adipose tissue leads to an increase in the secretion of tumor necrosis factor α (TNF-α), C-reactive protein (CRP), interleukin-6 (IL-6), monocyte chemoattractant protein -1(MCP-1), and other pro-inflammatory cytokines, which eventually leads to a chronic inflammatory state [26,27,28,29]. Due to the specific heterogeneity of adipose tissue, macrophages in adipose tissue also activate c-Jun amino-terminal kinase (JNK) and nuclear factor-κB (NF-κB) signaling pathways through autocrine and paracrine, which affects the surrounding tissues and organs, forming a vicious circle and aggravating the occurrence and development of obesity. Tumor necrosis factor superfamily regulates adipocyte inflammation and manipulates adipocyte function, thus promoting the occurrence of obesity and the development of complications [30]. Some researchers observed that the levels of TNF-α and leptin in obese patients increased, while the level of adiponectin decreased, and activated the RANK/RANKL/OPG system [31,32].

The role of the RANKL–RANK–OPG system in glucose and lipid metabolism has also been highlighted, in addition to the regulation of bone metabolism. RANKL is a type Ⅱ transmembrane protein, and RANK is its specific receptor; OPG is a pseudoreceptor of RANKL. RANK, RANKL, and OPG have well-established regulatory effects on bone metabolism. Moreover, RANK, RANKL, and OPG are also highly expressed in monocytes/macrophages, liver, muscle, kidney, heart, lung, vascular tissues, dendritic cells, and breast. OPG competitively binds to RANKL with RANK, thus blocking the mechanism of RANK. The binding affinity between OPG and RANKL is about 500 times higher than that of OPG and RANK. Therefore, OPG can prevent RANKL from binding to its receptor RANK. RANKL mainly exists in the Golgi apparatus. OPG affects the release of RANKL to the cell membrane and its extracellular transport process, leaving RANKL in the cells and thus reducing the binding of RANKL and RANK [33]. The activation of the RANK/RANKL/OPG signaling pathway is essentially a series of signal transduction reactions and biological effects after the binding of RANKL and RANK.

As a ligand of RANK, RANKL exists in two forms: soluble protein and transmembrane protein. RANK and RANKL genes are expressed in human liver tissues and pancreatic β cells, showing a close correlation with blood glucose control and obesity. The concentration of soluble RANKL is related to insulin resistance [34]. RANKL binds to its receptor RANK and activates the NF-κB pathway, which triggers the expression of pro-inflammatory cytokines [35], while obesity also activates the JNK and NF-κB signaling pathways [36]. NF-κB activation induces the activation of inflammatory signaling pathways, leading to insulin resistance and pancreatic β cell dysfunction [7,37], and also activates T cells, endothelial cells, and adipocytes [34]. However, there is an unknown interaction between bone and the immune system, which may lead to hepatic insulin resistance. RANKL can be used to link the interaction between immune activation, bone resorption, and obesity [34]. A previous study focusing on the epigenetic regulation of RANKL in obesity has reported that 73.86% of RANKL in the obesity group is unmethylated, while 80% RANKL in the non-obesity group is unmethylated. However, this study is retrospective and cannot determine the protein and gene expression levels of RANKL [36]. Our results suggested that obesity was related to the expression of the RANKL protein, RANKL mRNA, and RANK mRNA. Moreover, the expression of the RANKL protein in obesity patients was significantly higher than that in non-obesity patients. However, due to the large standard deviation and the small sample size, we shall further increase the sample size and conduct more in-depth research in the follow-up study. The expression of RANK mRNA in obesity patients was significantly higher than that in non-obesity patients, suggesting that RANK/RANKL/OPG was involved in the occurrence and development of overweight and obesity. Our findings may provide novel candidate genes and therapeutic targets for the management of obesity.

After the study, it was found that the expression of the RANKL protein in obese patients was significantly higher than that in non-obese patients, which was novel. Moreover, due to the large standard deviation and small sample size, we further increased the sample size and conducted more in-depth research in the follow-up study, so as to obtain the most complete conclusions and detailed results. To further clarify the relationship between the abdominal circumference of elderly obese patients and the expression of the RANKL protein, RANKL protein, gene, and OPG protein in white blood cells, and verify the influence of RANKL and RANKL proteins on obesity, a more careful thinking network was created. A thorough analysis suggested that RANK/RANKL/OPG participates in the occurrence and development of overweight and obesity. Our findings may provide new candidate genes and therapeutic targets for the treatment of obesity.

In conclusion, the RANK/RANKL/OPG system plays a vital role in the occurrence and development of obesity. Nowadays, there is an accumulating in-depth research on obesity due to the increasing global prevalence of obesity. The exploration of signaling pathways, cytokines, immune responses, and epigenetic modifications related to obesity is essential for disease outcome and treatment development. The RANK/RANKL/OPG system may be a promising target for obesity treatment.
